# The Adaptation of Pediatric Exercise Testing Programs to the Coronavirus/COVID-19 Pandemic

**DOI:** 10.1177/2150135120954816

**Published:** 2020-09-21

**Authors:** Adam W. Powell, Wayne A. Mays, Tracy Curran, Sandra K. Knecht, Jonathan Rhodes

**Affiliations:** 1 Department of Pediatrics, University of Cincinnati College of Medicine, OH, USA; 2 The Heart Institute, 2518 Cincinnati Children’s Hospital Medical Center, OH, USA; 3 Department of Cardiology, 1862 Boston Children’s Hospital, Boston, MA, USA

**Keywords:** cardiopulmonary exercise testing, COVID-19, severe acute respiratory syndrome coronavirus 2, personal protective equipment

## Abstract

**Objective:**

Response to the coronavirus/COVID-19 pandemic has resulted in several initiatives that directly impact hospital operations. There has been minimal information on how COVID-19 has affected exercise testing in pediatric patients.

**Design:**

A web-based survey was designed and sent to pediatric exercise testing laboratories in the United States and Canada. Questions were designed to understand the initial and ongoing adaptations made by pediatric exercise testing laboratories in response to COVID-19. Results were analyzed as frequency data.

**Results:**

There were responses from 42% (35/85) of programs, with 68% (23/34) of laboratories discontinuing all exercise testing. Of the 23 programs that discontinued testing, 15 (65%) are actively working on triage plans to reopen the exercise laboratory. Personal protective equipment use include gloves (96%; 25/26), surgical masks (88%; 23/26), N-95 masks (54%; 14/26), face shields (69%; 18/26), and gowns (62%; 16/26). Approximately 47% (15/32) of programs that typically acquire metabolic measurements reported either ceasing or modifying metabolic measurements during COVID-19. Additionally, 62% (16/26) of the programs that previously obtained pulmonary function testing reported either ceasing or modifying pulmonary function testing. Almost 60% of respondents expressed a desire for additional guidance on exercise laboratory management during COVID-19.

**Conclusions:**

Pediatric exercise testing laboratories largely closed during the early pandemic, with many of these programs either now open or working on a plan to open. Despite this, there remains heterogeneity in how to minimize exposure risks to patients and staff. Standardization of exercise testing guidelines during the COVID-19 pandemic may help reduce some of these differences.

## Introduction

In mid-December 2019, a novel strain of coronavirus (COVID-19) began in the Wuhan province and was noted to cause severe respiratory infections and began spreading rapidly around the world.^1^ After introduction into the United States, response to the COVID-19 pandemic resulted in several initiatives at the regional and national level to mitigate potential morbidity and mortality.^2^ Mirroring, and in many areas outpacing, the initiatives taken by governmental, societal, and business entities, the health care infrastructure has responded with a series of procedural, algorithmic, and material allocations designed to mitigate the morbidity and mortality associated with the COVID-19 pandemic. This includes treatment of positive COVID-19 patients,^3^ allocation of personal protective equipment (PPE),^4^ and triage based on urgency of medical and surgical procedures.^5,6^ Additionally, procedures that are associated with particulate aerosol have been categorized, and the risk to patient/health care workers has been quantified.^7,8^

These responses directly impact operations and methodology associated with cardiopulmonary exercise testing (CPET)/exercise testing. The exercise laboratory is in a unique position of risk as the aerosolization of particles from both symptomatic and asymptomatic patients could potentially infect patients, family, and staff.^9-11^ While guidelines have recommended the reduction or elimination of elective surgeries and procedures, minimal guidance has been issued for exercise testing. This has resulted in a lack of consensus on proper testing protocols, staffing models, and PPE use in exercise laboratories.

The primary aims of this study were to (1) better understand current practice patterns in pediatric exercise laboratories in the United States and Canada, (2) assess local and institutional management during the COVID-19 pandemic, and (3) investigate how centers are affected by the lifting of hospital restrictions for COVID-19.

## Materials and Methods

A 21-question online survey (REDCap) was designed and distributed to previous attendees of the Annual Clinical Exercise Testing and Therapeutics Symposiums (CETTS) in Cincinnati, Ohio, and programs on the Pediatric Exercise Testing and Therapy Network (PETTNet). The survey was distributed on May 13, 2020, and the collection of responses ended on May 21, 2020. A reminder email asking to complete the survey was sent on May 18, 2020, for those programs that did not respond to the initial email. Data were recorded regarding program location, changes to exercise laboratory staffing and operational protocols, current PPE use, changes to exercise testing protocols including deviations in measuring metabolic indicators of fitness, baseline pulmonary function testing, and noninvasive measures of cardiac output. More than one response per question was allowed, but only one completed survey was included per program. Lastly, a text box was added for the program to describe additional observations.

Survey responses were tabulated as frequency data where applicable (categorical data). Statistical analyses were performed using REDCap. This study was exempt from review by the Cincinnati Children’s Hospital Institutional Review Board.

## Results

**Figure 1.**
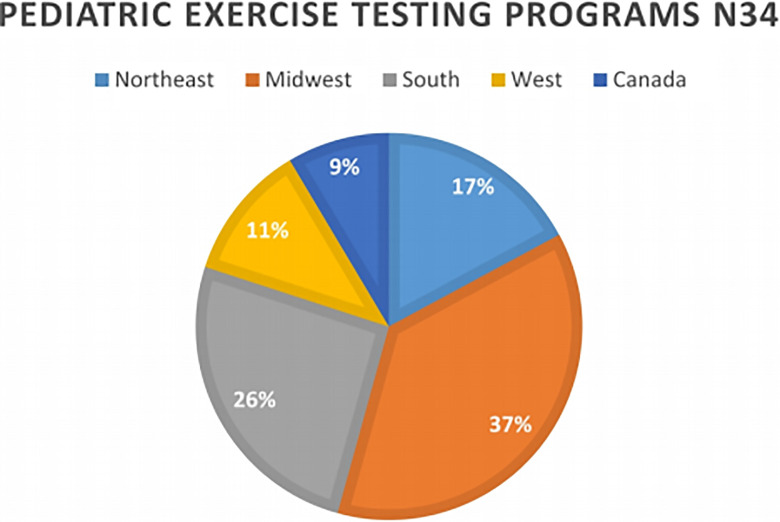
Pie chart demonstrating the breakdown of exercise laboratories that responded to the survey by geographic region. Northeast (CT, ME, MA, NH, NJ, NY, PA, RI, VT); Midwest (IL, IN, IA, KS, MI, MN, MO, NE, ND, OH, SD, WI); south (AL, AR, DE, DC, FL, GA, KY, LA, MD, MS, NC, OK, SC, TN, TX, VA, WV); west (AK, AZ, CA, CO, HI, ID, MT, NV, NM, OR, UT, WA, WY); Canada.

Surveys were completed by 35 (41%) of 85 programs that received a questionnaire. Of the 35 programs that completed the survey, 32 programs were located in the United States and 3 programs were located in Canada. Geographic regions where the responses originated are presented in Figure 1. Of the responding programs, 80% (28/35) were either from a tertiary or major academic medical center, 12% (4/35) were from regional hospitals, and 8% (3/35) identified as either free-standing or other.

Survey responses are listed in Table 1. The survey responders all reported that COVID-19 has led to major changes in the exercise laboratory, with the majority of programs stating that these changes occurred in mid-March (83%; 29/35). Among the programs surveyed, 66% (23/35) reported discontinuing all exercise testing for a period of time during the COVID-19 pandemic, 31% (11/35) continued testing but only for patients triaged by medical need, and 3% (1/35) did not alter testing protocols. Of note, the program that did not alter its testing protocols did not routinely perform metabolic measurements during exercise testing. Of the 23 programs that discontinued testing, 15 (65%) are actively working on triage plans to reopen the exercise laboratory, with the remaining 35% of exercise laboratories having no current plan to reopen. Staffing changes occurring for programs included 20% of programs mandating furlough for staff, 43% of programs rotating staff over multiple days/weeks, and 40% of programs mandating staff work from home. Only one program reported having an employee test positive or have symptoms of COVID-19 and three programs (9%) reported having to quarantine staff secondary to COVID-19 exposure.

**Table 1.** Results of Questionnaire Regarding Exercise Testing Laboratory Experiences and Changes Secondary to the COVID-19 Pandemic (More Than One Response Was Allowed).

Responding programs	N = 35
Hospital type	Academic/referral: 28 Regional: 4 Freestanding: 2 Other: 1
Timing of operational changes	Early March: 2 Mid-March: 29 Late March: 1 Early April: 2 Late April: 1
Modifications to exercise testing protocols	Yes: 27 No: 8
Changes to patients tested	Ceased all testing: 8 Patients triaged based on medical need: 11 Initially ceased testing but working on a plan to reopen: 15 Continued usual protocols without changes made: 1
Staffing modifications	Furlough: 7 Staff rotations: 15 Work from home: 14 No changes: 6
Symptomatic/confirmed COVID-19 in staff	None: 34 Yes: 1 (4 staff members involved)
Quarantined for COVID-19 exposure	None: 30 Yes, 1 staff member: 1 Yes, 2 staff members: 1 Yes, more than 5 staff members: 1
PPE used for exercise testing	None: 1 (their program remains closed) Gloves: 25 Surgical masks: 23 N-95 masks: 14 Face shields 18 Gowns 16 Other 7
Disinfectant used	Clorox Healthcare: 3 Caviwipes Bleach: 8 Caviwipes One: 10 Alcohol wipes: 6 Other: 18
Programs that performed metabolic measurements prior to COVID-19	Yes: 32 No: 2 No response: 1
Altered metabolic measurements for COVID-19	No: 18 Ceased obtaining metabolic measures: 8 Modified measurements: 7
Programs that obtained PFTs prior to COVID-19	Yes: 26 No: 9
Altered PFT measurements for COVID-19	No: 14 Ceased PFT: 9 Modified PFT: 7

Abbreviations: PFT, pulmonary function test; PPE, personal protective equipment.

There was a wide variation in the PPE used for exercise testing. Of the 26 programs that have either continued to conduct tests or have since restarted testing, PPE use included gloves (96%; 25/26), surgical masks (88%; 23/26), N-95 masks (54%; 14/26), face shields (69%; 18/26), and gowns (61%; 16/26), with 27% (7/26) reporting using other forms of PPE. One program reported not using PPE; however, they have remained closed and their answer likely reflects the fact that patients are currently not being tested at their center. Disinfectants used are summarized in Table 1.

There were 91% (32/35) of programs that reported typically acquiring metabolic measurements prior to the COVID-19 pandemic, and 47% (15/32) of these programs reported either ceasing or modifying metabolic measurements during COVID-19. Modifications of acquiring metabolic measurements included minimizing parents in the room, ensuring social distance, and adding antiviral/antibacterial filters to the end of the mouthpiece.

There were 74% (26/35) of programs that reported regularly obtaining pulmonary function testing prior to the COVID-19 pandemic, with 62% (16/26) of programs reporting they have either ceased or modified pulmonary function testing. Pulmonary function testing modifications included N-95 use during testing and using antiviral/antibacterial filters.

The survey had an open text field for the exercise laboratories to make general comments with several noteworthy responses given. These responses include three programs discussing the concern with contamination of the tubing involved with metabolic testing and needing to discuss this concern with the metabolic cart manufacturer. Additionally, five laboratories volunteered that they will require COVID-19 testing prior to having tests performed. Three laboratories volunteered that the infection control of their local hospital refused to supply or approve the use of N-95 masks for exercise testing. One program uses UV sterilization once a week in the laboratory. Lastly, 57% (20/34) voiced concern over the lack of guidance on this issue and/or hope for consensus on how to perform exercise testing during the pandemic.

## Comment

The novel coronavirus/COVID-19 pandemic has greatly affected many hospitals in the United States with over 1.5 million positive patients as of May 20. Pediatric centers have not been immune to the impact of COVID-19, which may even worsen with the emergence of pediatric multisystem inflammatory syndrome as a recently described pediatric sequela of COVID-19.^12^ While the impact of COVID-19 on pediatric patients with congenital and acquired heart disease is largely unknown, cardiac centers have altered local protocols as many of their patients are known to be at high risk for acquiring acute infectious viral illnesses.^13^ The CPET laboratory represents a troublesome combination of potentially high-risk patients in a testing environment that may lead to particle aerosolization.

The responses from this survey reflect how each program is seeking to protect patients and staff from COVID-19 complications through dramatically different protocols and plans. Over 65% of exercise testing programs stopped testing all patients at some point during spring 2020 despite the fact that only 9% of programs had an employee who tested positive, showed symptoms, or was knowingly exposed to someone with symptoms. There are likely several reasons for this. Given the lack of availability of PPE in many hospitals throughout the United States, many states mandated nonurgent testing to be postponed to a later date in order to preserve these potentially life-saving resources. Pediatric exercise laboratories across the country have largely followed these appropriate requests. Secondly, practice patterns likely shift in endemic areas with a high-virus prevalence, increasing the likelihood of transmission to the exercise testing staff, thus necessitating exercise laboratory closure. Lastly, the lack of standardized guidelines in the management of pediatric patients with congenital and acquired heart disease and COVID-19 likely plays a major role in the heterogeneity of the responses. The uncertainty related to the absence of guidelines was spontaneously disclosed by ∼60% of the respondents of the survey and is further demonstrated in the marked differences between programs in modifications to exercise testing protocols, reopening plans, and PPE utilization.

Personal protective equipment use has been a point of widespread concern in hospitals since the start of the current pandemic and will remain a major factor as exercise laboratories are reopened. This study has demonstrated a lack of consensus as to how programs are utilizing PPE to protect both staff and patients. While part of this may be secondary to geographic differences in COVID-19 distribution or governmental mandates on the limiting of nonurgent medical testing, another factor may also be the lack of recognition of what constitutes a “high-risk” procedure for particle aerosolization in the nonintensive care settings.^8-11^ The Center for Disease Control considers an aerosol-generating procedure to be a procedure that “creates uncontrolled respiratory secretions.”^9^ The European Society of Cardiology has recommended the avoidance of sputum-producing exercise in their guidance on providing cardiopulmonary rehabilitation during the COVID-19 pandemic.^11^ Pulmonary function testing, which was used in conjunction with exercise testing in 74% of our programs, is also felt to have the potential to induce secretions which may increase the risk of transmission.^10^ Despite the not inconsequential risk of aerosolization, only ∼50% of programs reported N-95 mask use, although this is somewhat skewed by three programs that were not allowed N-95 use by their local hospital. There does appear to be a consensus among programs performing tests on the use of a facial mask and gloves during testing, and two-thirds of programs also include facial shields and gowns to their standard PPE approach.

Lastly, while the administration of COVID-19 tests has been much discussed in the press, this will likely emerge as a major point of emphasis with the restarting of elective procedures and tests, including exercise testing. Preprocedural COVID-19 testing will also have additional importance as hospitals attempt to preserve PPE. Unfortunately, this survey was created prior to the implementation of widespread testing in the United States, so programs were not specifically asked in the survey whether COVID-19 testing is part of their reopening plan. Of note, there were five laboratories that volunteered that they currently require or plan to require a COVID-19 test prior to having an exercise test. This will take on particular importance as the country prepares for a second wave of infections. As the ability to test for COVID-19 improves, it may be vitally important to test all pediatric patients prior to aerosol-generating procedures secondary to the high rate of asymptomatic disease transmission in pediatric patients.^14,15^

This was a study based on voluntary survey completion from a cross section of national pediatric exercise testing laboratories, which results in several limitations. First, there was an overall small number of pediatric exercise testing programs that responded to the survey. While this will limit the ability of the study to make broad conclusions, it is worth noting that this represents a sizable response rate as there are not a large number of exercise testing facilities in the United States specializing in pediatric patients.^16^ Secondly, the COVID-19 pandemic has affected areas of the United States in different ways and at different times, resulting in a heterogeneity of responses to the outbreak based on geography. Thus, some of the conclusions from this survey may not be applicable to all areas of the United States or other countries, especially as this survey had a greater response rate from the midwestern and southern United States. Lastly, as this was a survey sent to selected programs based on affiliation with either the annual CETTS in Cincinnati, Ohio, or PETTNet programs, there is potential for sampling bias.

In conclusion, pediatric exercise testing programs have not been immune to the effects of the COVID-19 pandemic, with almost 66% of exercise testing facilities polled ceasing all testing at some point in time. There remains a great deal of heterogeneity between programs in the use of PPE and protocols for restarting routine exercise testing. With the wide differences in program responses to exercise testing protocols and PPE, standardized exercise testing protocols during COVID-19 testing may be very useful for programs regularly performing testing in this population.

## Abbreviations and Acronyms

CETTS: Annual Clinical Exercise Testing and Therapeutics Symposiums
CPET: cardiopulmonary exercise testing
PETTNet: Pediatric Exercise Testing and Therapy Network
PPE: personal protective equipment
